# Knowledge, attitude and practice of female university students regarding human papillomavirus and self-sampling in KwaZulu-Natal, South Africa: a cross-sectional survey

**DOI:** 10.1186/s12905-022-01634-z

**Published:** 2022-03-04

**Authors:** Miracle Tamaraebi Eche, Kerry Vermaak

**Affiliations:** School of Built Environment and Development Studies, University of KwaZulu-Natal, Durban, South Africa

**Keywords:** Cervical cancer, Cervical cancer screening, Human papillomavirus, Self-sampling, HPV test, KAP

## Abstract

**Background:**

Human papillomavirus (HPV) infection remains a major cause of cervical cancer. Screening practice in South Africa has remained persistently low, with the invasiveness of pelvic examination as a major barrier to screening. This occasions the need to assess women’s knowledge, attitude, and practice regarding HPV testing and self-sampling.

**Method:**

This is a cross-sectional quantitative study which enrolled 386 female students between the ages of 18 and 65 years at the University of KwaZulu-Natal, South Africa. Data was collected through a self-administered structured questionnaire, from February to March 2020. Data on participants’ socio-demographic characteristics, knowledge, attitudes and practices regarding HPV, HPV testing and self-sampling were obtained.

**Results:**

Out of the 386 respondents, 30.6% were unaware that HPV can be transmitted through unprotected sex, only 25.1% knew about the availability of HPV vaccines in South Africa, 16.1% knew that the vaccines are accessible for free, while 79.0% were oblivious to the asymptomatic nature of HPV infection. Furthermore, a vast majority (95.8%) had never heard about self-sampling while only 1.0% had undergone HPV testing prior to this study. Although 52.9% knew that HPV testing could prevent cervical cancer, it did not positively impact screening practice. However, 57.7% of participants were willing to undergo future screening if allowed to self-sample.

**Conclusion:**

Self-sampling is a more acceptable means of sample collection compared to pelvic examination. Therefore, encouraging self-sampling and providing self-sampling kits will aid increased screening participation and address certain barriers associated with HPV testing. Awareness and educational campaigns about HPV and its causative relationship with cervical cancer will occasion better attitude towards screening participation.

## Background

Human papillomavirus (HPV) is one of the leading causes of cancer of the vulva and vagina, and cervical cancers [1]. HPV can lie dormant and unnoticed for a long period, and women could develop antibodies against it [2]. However, persistent high-risk HPV infections have a great likelihood of developing into precancerous lesions which may metamorphose into full blown cancers [1]. Globally, the high-risk HPV types 16 and 18 are found to be most prevalent causes of cancer, closely followed by other high-risk types (31, 39, 51, 52, 56, 58, and 59) [1]. HPV has been recorded to be most prevalent among younger women (16–22 years) [3]. Research has also shown a similar prevalence rate among all age groups of women in several low-income countries (LICs) in Africa and Asia [4].

In South Africa, the first research to ascertain the rate of HPV prevalence was conducted in KwaZulu-Natal from March 2004 to May 2007 [5]. The study enrolled 224 sexually active HIV-negative women between the ages of 14–30 years, from whom blood and cervical samples were obtained. The general HPV prevalence rate was 76.3% (171/224), and 70.5% for women in rural areas [5]. In another study, the burden of HPV among women was shown to weigh heavily on both rural and urban areas in KwaZulu-Natal [6]. A similar prevalence rate (74.6%) was recorded in a study carried out in Gauteng province in 2013, where 1 472 women attending five urban and peri-urban public health clinics were enrolled [7].

HPV infections can be prevented from developing into full blown cancer if they are detected early and treated [8, 9]. Early detection has occasioned decline in incidences of cervical cancer and related deaths; with women who receive prompt treatment for precancerous lesions having an almost 100% 5-year chance of survival [10]. Administering HPV vaccines to males and females is another mode of prevention. South Africa’s policy for prevention and control of cervical cancer provides for the administration of HPV vaccines and propagation of awareness on HPV prevention through dual protection and other safe sex practices [11].

Another cervical cancer preventive mode is HPV testing because it has a better negative predictive value (NPV) and is more sensitive in the detection of precancers and cancer than cytology-based screening. Thus, HPV testing allows for longer screening intervals especially in poor-resource settings. [12]. However, most women do not undergo screening or get screened early enough, and vaccination rates remain persistently low [10] which necessitates continued HPV screening. Nevertheless, self-sampling for HPV testing can occasion greater coverage and encourage increased participation in screening among women.

Self-sampling is an innovative procedure which allows women to privately collect their own cervical samples, whenever, and wherever they deem convenient [13]; these samples are thereafter sent to the lab for testing [14]. Follow-up by the health system becomes necessary in cases where the tests results are positive [14]. Studies conducted globally show that self-sampling is an adequate mode of sample collection for HPV testing [15–18] and that tests done on self-collected samples are as accurate as that of clinically-collected samples [19–21]. Thus, increasing the likelihood of greater screening coverage in low-income areas [22].

### Cost-effectiveness of Self-sampling for HPV testing

Self-sampling for HPV testing has been found to be a cost-effective alternative to clinic-based cervical screening. This was evidenced in a recent study which assessed the cost-effectiveness of repeated HPV self-sampling in comparison with cytology-based (pap smear) screening in the detection of cervical intraepithelial neoplasia grade 2 or more (CIN2+). The study involved a cost-effectiveness analysis (CEA) carried out on data from a randomized clinical study which had been previously published. The clinical study enrolled 36,390 women between the ages 30 to 49 years. Participants were randomized either to undergo repeated HPV self-sampling of vaginal fluid (n = 17,997) or to undergo midwife-collected Pap smears for cytological testing (n = 18,393). Self-sampling for HPV testing resulted in "1633 more screened women and 107 more histologically diagnosed CIN2 + at a lower cost vs. midwife-collected Pap smears (€ 229,446 vs. € 782,772)" [23].

### Review of literature on knowledge, attitude and screening practice involving self-sampling

There remains a paucity of studies on the knowledge, attitudes, and practices regarding HPV and self-sampling for HPV testing; thus, literature from studies carried out in more developed countries has been included in this review.

### Attitude towards self-sampling

Notwithstanding the positive impact of self-sampling on HPV testing, it is important to have an understanding of the perceptions and attitude of women regarding this method of cervical sampling in order to successfully incorporate it into national screening programs [22].

Studies carried out globally in diverse settings suggest that most women consider self-sampling an acceptable method of sampling for HPV testing [15, 16, 24, 25]. In a study carried out in Cape Town, South Africa, involving 822 women, most participants exhibited positive attitudinal disposition to self-sampling [22]. The majority of the sample population (93.6%) indicated that they did not feel embarrassed, while 89.4% stated that they experienced no discomfort during self-sampling. Participants showed a readiness to perform self-sampling (93.9%); however, some preferred to do it at the clinic due to cost of taking the samples back to the clinic. In spite of their positive attitude, 64.7% of participants expressed more confidence in clinically-collected samples than self-collected samples. Some expressed anxieties over the quality of samples they collected, contamination and fear of the samples drying out [22].

In a study carried out in Brunei, 174 non-attendees to cervical screening were enrolled, out of which 97 also participated in HPV self-sampling. Those who participated in self-sampling generally had positive responses regarding the use of the self-sampling kits. Most stated (94.8%) that the instructions were clear, 93.8% indicated that the swab was easy to perform, while 91.7% considered it to be more convenient than the Pap test. Furthermore, 92.8% expressed confidence that they correctly obtained their samples, 94.8% expressed their willingness to self-sample in the future, while 93.8% stated that they would recommend self-sampling to other women [26].

Similarly, in a Belgian study, 515 women aged 25–64 years were enrolled in the VALHUDES trial from five colposcopy clinics. A vast majority (93%) of the participants confirmed that self-sampling could aid increased participation among under-screened women, 95% stated that the self-sampling instructions were clear. procedure was easy, and expressed confidence in having correctly performed the procedure. However, 44% of the participants expressed more confidence in the effectiveness of a clinician-collected sample over a self-collected sample while a proportion of women considered self-sampling with cotton swabs or plastic brushes unpleasant. Regardless, more women (57%) expressed their willingness to self-sample in the future than those who indicated that they would prefer their samples to be obtained by a clinician [27].

Furthermore, in a study carried out in India, Nicaragua and Uganda, a total of 19,340 women were screened, and out of those who carried out self-sampling, 75% considered the process easy, 52% reported initial anxiety over getting hurt but found it painless, while 24% were concerned about the quality of the self-collected sample [15]**.** Most preferred self-sampling in the clinic rather than at home because the clinic provided a cleaner, more private environment; furthermore, samples could be easily and promptly handed over for testing and treatment received, where necessary [15].

However, in an Australian study, only 34% of the 3,000 participants expressed preference for self-sampling, majority of whom had not undergone Pap smear testing in over 3 years. Reasons given for self-sampling preference include: convenience, ease, privacy, and that it is less embarrassing and time-consuming. Fifty-seven percent (57%) preferred to have their samples collected by a clinician while 8% were uncertain [28]. Reasons for aversion to or uncertainty about self-sampling include: confidence in the skill of clinicians, anxieties over accuracy/reliability of self-collected samples and doubts regarding their ability to do it right [28].

It has been revealed that there are some existing barriers to collecting self-samples at home; nevertheless, many healthcare facilities in developing countries lack adequate human resources to obtain the cervical samples of everyone who need to be screened [22]. In this case, self-sampling in the healthcare facility can significantly aid in the reduction of overcrowding, shortage of skilled personnel and prolonged waiting times in health centres which is associated with clinical collection of samples [22, 29]**.** These anxieties and misconceptions over self-sampling for HPV screening can be effectively tackled by dissemination of information [28], proper counselling and health education [22]. Furthermore, it will be beneficial to carry out pilot studies to implement the self-sampling method prior to its adoption and integration into screening programs [22].

### Current self-sampling practices

Self-sampling can potentially address most of the known screening limitations among under-served and unparticipating women, [13, 14, 30, 31]. Thus, occasioning increased screening participation among unparticipating women (Duke et al., 2015). These limitations include: gender of person carrying out screening [32–34]; Embarrassment, pain and/or dislike associated with pelvic examination [33, 35–37]. Based on a review of studies that were conducted in predominantly industrialised settings, Gupta et al. (2018) concluded that women failed to screen because of: their prior experiences with conventional sampling techniques which they perceived to be negative due to their cultural background/beliefs, pain and discomfort associated with conventional sampling, personal history of abuse, poor knowledge, low perceived susceptibility, as well as socio-economic challenges associated with attending conventional clinical appointments [38]. Self-sampling is considered a solution to these barriers that impede participation in cervical screening. Hence, it has been reported to increase cervical screening practice [38].

In a study conducted in India, Nicaragua and Uganda, involving 19,340 women, 90% presented self-collected samples; 78% preferred self-collection of samples to having a clinician collect same [15]. In another study from Nicaragua, the acceptability of self-sampling for HPV testing was significantly higher than clinical collection. The majority (81.1%) of participants expressed willingness to self-sample in the future because they considered it comfortable, less painful, less invasive and less embarrassing [39].

In a Canadian study, 88.8% of the women who self-collected their samples reported that the process was satisfactory; 15.5% of which had never been screened or had irregular screening practice. The provision of self-collection kits gave rise to their willingness to participate in screening. It is worthy of note that, though the response rate was poor, cervical cancer screening improved by 15.2% to 67.4% where self-sampling was offered, as opposed to a 2.9% improvement in areas where educational campaigns only were carried out [40].

On the other hand, a study in Mexico, involving women of low socio-economic standing, recorded a 74.6% response rate to self-sampling [41]. Another study in rural Mississippi recorded a 64.7% response to self-sampling—80.5% of which returned their samples; as opposed to the 35.3% who opted for Pap smear, only 40.5% of which were present at their clinic appointment. The number of under-screened women who self-sampled for HPV testing were almost 4 times greater than those who opted for Pap smear (78.4% vs 21.5%) [42]**.** In these studies, however, nurses went to the households of the participants and assisted them with paperwork and sample retrieval. The greater level of participation in these studies is attributable to the specialized care offered to participants. Nevertheless, this is not always practicable [40].

As stated earlier, there is a dearth of studies on women’s knowledge, attitudes, and practices regarding HPV and self-sampling for HPV testing especially in Africa where little or nothing has been done. In sub-Saharan Africa, a study which focused primarily on assessing young people’s knowledge, attitude and practices concerning cervical cancer in five provinces of Zimbabwe reported little on participants’ knowledge about HPV. The study which enrolled 751 males and females from high schools and universities reported that less than half (47%) of the participants knew about HPV transmission and prevention [43].

It is therefore necessary to encourage self-sampling and propagate knowledge about its possibility, accuracy, and usefulness [13] especially in Africa. However, in order to successfully achieve greater participation in screening, the knowledge, attitudes and willingness of women must be assessed.

The current study was designed to assess young women’s knowledge, attitude, and practice regarding HPV and self-sampling for HPV testing. Such an assessment is necessary to aid interventive programs and policy initiation. This is because greater participation cannot be achieved if women lack the knowledge to seek or have an unfavourable attitude to screening. Previous studies assessed the prevalence of HPV, adequacy of self-sampling, and the accuracy of tests conducted using self-collected samples. This study is particularly relevant as there is currently no other study, to the best of our knowledge, which assesses the knowledge, attitude and practice of female university students regarding HPV and self-sampling for HPV testing in KwaZulu-Natal, South Africa and in Africa at large.

## Methodology

### Study design and setting

This cross-sectional study was conducted in the University of KwaZulu-Natal; one of the leading institutions in South Africa with five campuses (Edgewood, Medical School, Howard College, Westville and Pietermaritzburg). Howard College Campus was purposively selected for inclusion, as the location made planned systematic data collection feasible. Furthermore, obtaining data from students in colleges that are health-related would not have truly reflected or achieved the aim of this study as they would typically possess better knowledge, have better attitude towards HPV testing and self-sampling, and ultimately engage in better preventive practices [31, 44, 45].

### Gate keeper’s permission and ethical approval

The gatekeeper’s permission to conduct this study was obtained from the University of Kwazulu-Natal. This study was approved by the Humanities and Social Sciences Research Ethics Committee (HSSREC) of the University of KwaZulu-Natal, with Protocol Reference Number HSSREC/00000563/2019.

### Study population

The study was conducted within a period of 12 months. After obtaining the required gatekeeper’s permission and ethical approval, data was collected from February to March 2020 using self-administered structured questionnaires. A total of 386 registered female students between the ages of 18–65 years were enrolled from undergraduate and postgraduate levels of the College of Humanities, at the Howard Campus of the University.

### Sample size

Sample size was calculated using Sample Size Calculators on the UCSF Clinical and Translational Science Institute website [46]. The following parameters were used: confidence level (CL) of 95%, expected proportion (P) of 0.5, and total width of confidence interval (W) of 0.1. This gives a sample size of 384. This sample size was increased by 10% to allow for non-response.

### Recruitment process

The data was collected by the principal investigator, participants were chosen using a systematic random sampling technique. Using the lecture timetable for Howard College, forty slots were systematically selected, from a random starting number. The researcher stood outside the venue and approached potential participants until 12 questionnaires had been accepted by willing participants after they were duly informed about the study, and had signed the informed consent form. The questionnaires carried relevant instructions which aided self-administration by participants, ease of completion and uniformity. The questionnaire ensured the anonymity of participants, authenticity of information and protection of confidentiality at all times. The data was collected in this manner until the necessary sample size was reached, then data collected was analysed.

### Response rate

A total of 425 questionnaires were handed out, out of which 417 questionnaires were returned while 8 were unreturned. Out of the returned questionnaires, 31 were incompletely filled while 386 were completely filled out—thus, the response rate was 90.8%.

### Inclusion and exclusion criteria

Before the data collection tool was given to the participants, the purpose of the study was explained to them and their informed consent duly obtained. Only willing female students within the specified age bracket (18–65 years) and in the College of Humanities were included. Female students who were not in the College of Humanities, were unwilling to participate, below and above the specified age, and unable to respond to the questionnaire were excluded.

### Data collection

Participants’ socio-demographic information—such as age, race, educational level, sexual relationship status, sexual activity, contraceptive use, and family history of cervical cancer—were obtained. Data relating to participants’ knowledge, attitude, and practice regarding HPV, HPV testing and self-sampling were also obtained. Knowledge was construed as the respondents’ state of awareness about HPV infection, its relationship with cervical cancer, asymptomatic nature, mode of transmission and prevention, vaccine availability and accessibility, and HPV testing and self-sampling. Participants who provided correct answers to the questions in the knowledge section of the questionnaire were considered knowledgeable. Attitude was construed as the respondents’ perceptions (whether positive or negative) about the disease, and the practice of HPV testing and willingness to self-sample for the test.

### Data analysis process

Data collected was analysed using the Statistical Package for the Social Sciences Statistics (SPSS) Version 25. Mean ± SD or median (interquartile range) was used to express continuous variables of participants. The chi-square test or fisher’s exact test was used to compare categorical variables which were expressed as proportions where appropriate. Where the significance level (p value) was below 0.05, the results were considered statistically significant.

### Theoretical framework

The theoretical framework used for this study is the Health Belief Model (HBM0. The HBM is one of the oldest concepts that seek to describe the health behaviour of individuals [47]. It was first propounded in the 1950s and has now become one of the most commonly used and ‘recognized conceptual frameworks of ‘health behaviour’ globally [48, 49]. This model was first propounded to unravel the rampant issue of non-participation in programs aimed at preventing and detecting illnesses [49, 50]. It was later broadened to analyse the response of individuals to symptoms [51], their reactions when they are diagnosed of a disease, and most importantly, how they stick to a prescribed medical treatment plan [52].

The constructs of the health belief model are hinged on two theories: ‘perceived threat’ and ‘behavioural evaluation’. Perceived threat comprises of two major beliefs—perceived susceptibility to disease and perceived seriousness of the disease. While behavioural evaluation comprises of two beliefs—benefits of a prescribed health regimen and the barriers or limitations of undergoing such prescribed action [53, 54].

The four major constructs are: perceived susceptibility, seriousness, benefits and barriers [48, 55]. The health belief model posits that an individual will take preventive measures if he/she is of the belief: that they are prone to a certain disease (susceptibility); that the impact the disease will have on them is severe (severity); that the perceived susceptibility could result in change in a person’s health behaviour if they believe that the action prescribed to combat or reduce the disease is effective (benefits); and though there are possible barriers or costs that may hinder or limit their involvement in the prescribed actions, the belief that the advantages of the prescribed action outweighs the costs (costs/barriers) [47].

Generally, the results of quantitative analysis of the four major constructs of the HBM show that they sufficiently predict health behaviours, though with minimal effects. It is therefore advised that these constructs be combined to produce a greater effect [54]. This model has been effective in predicting health-associated behaviours and in setting up intervention schemes, including cancer screening behaviours [52].

However, it has some limitations:

1. Actively weighing the costs and benefits of a health behaviour does not always influence habitual behaviours like smoking, although this limitation would not apply to screening tests which occur much less frequently.
2. The model recognizes an individual’s desire to avoid problems but fails to consider factors that can enable them maintain preventive behaviour in the long run [52].
3. Though this model recognizes predictors that can possibly promote ‘adherence to medical regimens’, it contributes little to understanding the factors that causes such adherence [47].

These constructs of the HBM were used in the development of the knowledge and attitude questions in the data collection tool used for this study.

### Determination of participants’ knowledge and attitude

Participants’ responses to the questions in the knowledge section of the questionnaire were scored to determine the knowledge level. Scores ranging from 0 to 49% were deemed poor knowledge while scores ranging from 50% and above were considered good knowledge. The attitude of participants to HPV self-sampling was construed using their responses to questions in the questionnaire regarding their willingness to undergo HPV testing if allowed to self-sample.

## Results

### Socio-demographic characteristics

Table 1 is a presentation of the socio-demographic characteristics of the study participants. A total of 386 participants were enrolled in this study, majority of whom were Black (94.0%) and between the ages of 18–24 (85.2%). All participants were literate and enrolled at various levels in the university. A large percentage (93.5%) were unemployed, as study was their full-time occupation. Over half (55.4%) were in a sexual relationship, while 44.5% were not sexually active at the time the study was carried out. A little over half of the participants (51.0%) had one sexual partner within the preceding year, 14.5% had multiple sexual partners, while 34.4% had none. Amongst the participants who are in a sexual relationship, 53.7% stated that they always use contraceptives, while 46.2% stated that they use contraceptives sometimes. Furthermore, when asked what kind of contraceptives they use, 63.5% of those in a sexual relationship stated condoms, 14.5% oral contraceptives, while 21.9% stated that they use other kinds of contraceptives. Nearly three quarters (73.5%) had not tested for STI within the past six months, while 26.4% had tested. Only 6.9% of participants had treated STI in the past six months, while 93.0% had not. Majority (94.0%) of the participants had no family history of cervical cancer, as opposed to the 5.9% who had.

**Table 1:** Participants’ socio-demographic characteristics

Characteristics	Frequency (n)	Percentage (%)
**Ethnic group**		
African	363	94.0
Indian	10	2.6
White	5	1.3
Coloured	8	2.1
**Age**		
18–24	329	85.2
25–30	50	12.9
31 and above	7	1.8
**Highest level of education obtained**		
Diploma	11	2.8
Bachelors/Honors	169	43.7
Masters	14	3.6
Matric/others (Honours/Btech/N6)	192	49.7
**Employment status**		
Employed/self-employed/freelance	23	6.4
Unemployed	361	93.5
**Are you in a sexual relationship?**		
Yes	214	55.4
No	172	44.5
**Number of sexual partners in the past year**		
None	133	34.4
One	197	51.0
Two or more	56	14.5
**If sexually active, how often do you use contraceptives?**		
Always	115	53.7
Sometimes	99	46.2
**What kind of contraceptives do you use?**		
Condoms	136	63.5
Oral contraceptives	31	14.5
Others	47	21.9
**Have you tested for STI in the past 6 months?**		
Yes	102	26.4
No	284	73.5
**Have you been treated for STI in the past 6 months?**		
Yes	27	6.9
No	359	93.0
**Family history of cervical cancer**		
Yes	23	5.9
No	363	94.0

### Participants’ knowledge regarding HPV and self-sampling

Table 2 shows the frequency and percentage distributions of respondents’ responses to questions that assesses their knowledge about HPV and self-sampling.

**Table 2:** Table showing frequency and percentage of respondent’s response to knowledge items on the questionnaire

Question	Frequency (n)	Percentage (%)
**Human Papillomavirus (HPV) infection can be contracted through unprotected sexual intercourse**		
Yes	118	30.6
No	21	5.4
I don’t know	247	64.0
**Are there vaccines for protection against Human Papillomavirus (HPV) infection?**		
Yes	101	26.2
No	13	3.4
I don’t know	272	70.5
**Is the HPV vaccine available in South Africa?**		
Yes	97	25.1
No	7	1.8
I don’t know	282	73.1
**Is the HPV vaccine free of charge?**		
Yes	62	16.1
No	23	6.0
I don’t know	301	78.0
**Can HPV infection be treated?**		
Yes	125	32.4
No	12	3.1
I don’t know	249	64.5
**Have you heard about HPV self-sampling?**		
Yes	16	4.1
No	370	95.8
**You can have HPV for many years and not show symptoms**		
Yes	81	21.0
No	9	2.3
I don’t know	386	76.7
**You don’t need a doctor to collect your cervical sample for HPV testing, you can collect it yourself**		
Yes	49	12.7
No	34	8.8
I don’t know	303	78.5

#### Knowledge about HPV

In response to the question whether HPV can be contracted through unprotected sexual intercourse, majority (64.0%) stated that they had no idea, 30.6% rightly said yes, while 5.4% said no. In relation to the question as to whether there were vaccines for protection against HPV, 70.5% of participants had no idea, 26.2% rightly said yes, while 3.4% said no. When asked whether HPV vaccines are available in South Africa, 73.1% had no idea, 25.1% rightly said yes, while 1.8% said no. Regarding whether the HPV vaccine is free of charge, 78.0% had no idea, 16.1% rightly said yes while 6% said no. As to whether HPV infections can be treated, 64.5% had no idea, 32.4% rightly said yes, while 3.1% said no. As to whether HPV infection can be asymptomatic, 76.7% had no idea, 21.0% rightly said yes, while 2.3% said no.

#### Knowledge about HPV self-sampling

A vast majority (95.8%) of participants had never heard of HPV self-sampling while only 4.1% had heard of HPV self-sampling. Majority (78.5%) had no idea that self-sampling was possible, 12.7% rightly said self-sampling is possible, while 8.8% said no.

Table 3 shows the factor loading analysis and reliability test results for the knowledge section of the questionnaire. Based on the factor analysis, the questions on knowledge were categorised into two: knowledge about HPV and knowledge about HPV self-sampling.

**Table 3:** Factor loading on knowledge regarding HPV and HPV self-sampling among female students at the University of KwaZulu-Natal

Categories and items	Factor loading	Alpha
**1. Knowledge about HPV**		
Human Papillomavirus (HPV) infection can be contracted through unprotected sexual intercourse	0.69	0.86
Are there vaccines for protection against Human Papillomavirus (HPV**)** infection?	0.69	
Is the HPV vaccine available in South Africa?	0.76	
Is the HPV vaccine free of charge?	0.73	
Can HPV infection be treated?	0.64	
You can have HPV for many years and not show symptoms	0.71	
**2. Knowledge about HPV self-sampling**		0.73
Have you heard about HPV self-sampling?	0.74	
You don’t need medical personnel to collect your cervical sample for HPV testing, you can collect it yourself	0.79	

Table 4 shows the descriptive statistics for the various knowledge groups. It also shows the mean score of participants on knowledge about HPV and knowledge about HPV self-sampling.

**Table 4:** Descriptive statistics for knowledge categories

	Number of items	Mean ± SD
Knowledge about HPV	6	14.75 ± 3.77
Knowledge about HPV self-sampling	2	4.99 ± 1.00

SD = standard deviation

Table 5 depicts the use of Pearson’s correlation coefficient to ascertain the relationship between the different knowledge groups. The results show a significant positive relationship between knowledge about HPV and knowledge about HPV self-sampling.

**Table 5:** Pearson correlations coefficient results for the different knowledge categories

	1	2
1. Knowledge about HPV	1.00	
2. Knowledge about HPV self-sampling	0.50**	1.00

**Significant at 0.01 level

### Differences in participants’ knowledge scores with regards to sexual relationship status

Table 6 is a presentation of the mean knowledge score of the study participants with regards to their sexual relationship. The result shows no significant difference in the knowledge score of participants who are sexually active and those not sexually active with reference to all the knowledge categories (p > 0.05).

**Table 6:** Difference in the mean scores of knowledge categories with regards to sexual relationship status of participants

Measure	Sexual relationship	Mean ± SD	95% CI for Mean		T-value	p-value
			Lower boundary	Upper boundary		
Knowledge about HPV	Yes	14.49 ± 3.84	− 1.36	0.16	− 1.56	0.21
	No	15.09 ± 3.66	− 1.35	0.15		
Knowledge about HPV self-sampling	Yes	4.87 ± 1.04	− 0.46	− 0.07	− 2.65	0.13
	No	5.13 ± 0.89	− 0.46	− 0.07		

### Differences in participants’ knowledge scores with regards to family history of cervical cancer

Table 7 is a presentation of the mean knowledge score of the study participants with respect to their family history of cervical cancer. Between the participants who had a family history of cervical cancer and those who did not, it was observed that there was no significant difference in the knowledge score with regards to “knowledge about HPV” (P > 0.05). There was significant difference (p < 0.05) in knowledge score of participants who do not have a family history of cervical cancer (5.00 ± 0.97) and those who have a family history of cervical cancer (4.74 ± 1.18) with regards to knowledge about HPV self-sampling.

**Table 7:** Difference in the mean scores of knowledge categories with regards to participants’ family history of cervical cancer

Measure	Family history of cervical cancer	Mean ± SD	95% CI for Mean		T-value	p-value
			Lower boundary	Upper boundary		
Knowledge about HPV	Yes	15.43 ± 3.06	− 0.86	2.33	0.91	0.12
	No	14.70 ± 3.81	− 0.64	2.11		
Knowledge about HPV self-sampling	Yes	4.74 ± 1.18	− 0.68	0.15	− 1.24	0.02
	No	5.00 ± 0.97	− 0.78	0.26		

### Differences in participants’ knowledge scores with regards to their HPV test practice

Table 8 is a presentation of the mean knowledge score of the study participants with respect to whether they had ever tested for HPV. Between the participants who had done HPV test in the past and those who had not, there was no significant difference in the knowledge score with regards to knowledge about HPV, and knowledge about HPV self-sampling (P > 0.05).

**Table 8:** Difference in the mean scores of knowledge categories with regards to whether participants had ever tested for HPV

Measure	Practice: Ever had HPV test	Mean ± SD	95% CI for Mean		T-value	p-value
			Lower boundary	Upper boundary		
Knowledge about HPV	Yes	9.25 ± 3.40	− 9.25	− 1.88	− 2.97	0.35
	No	14.81 ± 3.73	− 10.94	− 0.19		
Knowledge about HPV self-sampling	Yes	4.00 ± 0.82	− 1.96	− 0.03	− 2.03	0.64
	No	5.00 ± 0.98	− 2.28	0.29		

### Participants’ perception regarding HPV and HPV testing

Table 9 shows the percentage distribution of participants’ response to questions on their perception of the severity of HPV and the benefits of HPV testing. When asked whether untreated HPV infection causes cervical cancer, 35.7% of participants said yes, 4.1% said no, while 60.1% stated that they were not sure. When asked whether they believed HPV testing could prevent or reduce their chances of getting cervical cancer, 56.9% of participants said yes, 5.6% said no, while 37.3% stated that they were not sure.

**Table 9:** Participants’ response to questionnaire items under the attitude section

Question	Frequency (n)	Percentage (%)
**The Human papillomavirus (HPV) causes cervical cancer, if not treated**		
Yes	138	35.7
No	16	4.1
Not sure	232	60.1
**HPV testing can prevent or reduce my chances of getting cervical cancer**		
Yes	220	56.9
No	22	5.6
Not sure	144	37.3

### Participants’ HPV testing practice

Table 10 shows the response of participants to questionnaire items under the practice section. Out of the 386 participants, only 4 (1.0%) had ever tested for HPV while a vast majority (98.9%) had never had one; everyone who had tested stated that they were informed of the result. When asked whether they were willing to undergo routine HPV testing if they are allowed to self-sample, 57.7% of participants said yes, 5.1% said no, while 37.0% stated that they were not sure. When asked whether they were willing to undergo routine HPV testing if they are not allowed to self-sample, 37.5% of participants said yes, 20.7% said no, while 41.7% stated that they were not sure.

**Table 10:** Table showing frequency and percentage of respondent’s response to practice items on the questionnaire

Question	Frequency (n)	Percentage (%)
**Have you ever tested for HPV?**		
Yes	4	1.0
No	382	98.9
**If yes, were you informed of the result?**		
Yes	4	100
No	0	0
**I am willing to undergo routine HPV testing if I can collect my samples myself**		
Yes	223	57.7
No	20	5.1
Not sure	143	37.0
**I am willing to undergo routine HPV testing even if I am not allowed to collect my samples myself**		
Yes	145	37.5
No	80	20.7
Not sure	161	41.7

## Discussion

Based on the assessment of participants’ knowledge about HPV, this study revealed that 30.6% of participants were unaware that HPV can be transmitted through unprotected sex; this is less than half of the percentage (64.0%) recorded in a Kenyan study [56]. Furthermore, just over a quarter (26.2%) of participants in this present study knew that there are HPV vaccines, and 25.1% knew about the availability of the HPV vaccines in South Africa, while less than one-fifth (16.1%) knew that these vaccines can be accessed for free in South Africa. This is much lower than the 54.4% who knew about the availability of HPV vaccines as recorded in an Indian study [31]. Over two-third (67.6%) of participants in the current study were unaware that HPV infections can be treated, while a vast majority (79.0%) were oblivious to the asymptomatic nature of HPV infection—which is lower than the 49.5% reported in the Indian study [31]. Flowing from the above, it is apparent that the knowledge of participants regarding HPV is generally low. The resultant effect is that participants are less likely to understand their susceptibility to HPV infection and less inclined to undertake preventive measures against the disease, such as screening. This amplifies the need for educative campaigns and dissemination of proper information about the disease.

Regarding participants’ knowledge about HPV self-sampling, the results from this study indicate that a vast majority (95.8%) of participants had never heard of HPV self-sampling prior to this study. Over three-fourth (78.5%) were unaware of the possibility of self-sampling, while 8.8% stated that self-sampling for HPV test is impossible. It is clear from the above that participants’ knowledge about HPV self-sampling is much lower than their knowledge about HPV. This further amplifies the need for proper education on the possibility, benefits, and accuracy of self-sampling in order to encourage greater screening participation.

The knowledge level of participants who were sexually active, at the time of the study, was compared to that of sexually inactive participants. Results show no significant difference in participants’ knowledge score on both knowledge categories. This is attributable to the fact that the participants are all university students, and would generally have similar knowledge level, their sexual relationship status notwithstanding.

Results show that participants without a family history of cervical cancer showed better knowledge regarding HPV self-sampling than those who had a family history of cervical cancer. This illustrates that there is a scarcity of knowledge about self-sampling and the connection between HPV and cervical cancer even among people with a family history of cervical cancer. However, this may be due to the fact that the number of participants with a family history of cervical cancer is much fewer (n = 23) than those without a family history of the disease (n = 363). Thus, the result may not accurately reflect the actual knowledge level.

The knowledge score of participants who had tested for HPV prior to the study was compared with that of those who had never tested. Results show that those who had previously tested for HPV were more likely to have better knowledge of cervical cancer than those who had never undergone HPV test. It is arguable that better knowledge about the disease results in better screening practice; the more an individual knows about the disease, the more they undertake preventive actions against it. Conversely, it may also be argued that they have better knowledge because they were informed about cervical cancer and its relationship with HPV when they were counselled for the test, and not necessarily that they underwent screening because they had better knowledge.

Regarding participants’ perceived severity of HPV infection, only 35.7% of the sample population in this study were aware that untreated HPV infection can cause cervical cancer, which is lower than the 55.0% reported in a study carried out in Pakistan [57]. The higher percentage recorded in the Pakistani study can be attributed to the fact that almost two-thirds (64.1%) of the participants were health science students who would definitely possess better knowledge about the disease than the participants in the current study. The percentage of students in the present study who stated that untreated HPV infection causes cervical cancer is relatively close to the 30.6% who cited HPV as a possible cause/risk factor of cervical cancer in this study. It is clear that the percentage of participants who understand the severity of HPV infection is a lot lower than the percentage who understand the severity of cervical cancer. This may be attributed to the dearth of knowledge about the causative relationship between HPV and cervical cancer, and also the fact that majority of participants in the present study do not believe they are susceptible to either cervical cancer or HPV. Similarly, though about one-third (30.6%) of participants understood the severity of HPV, the percentage of participants who had undertaken screening practice is much lower (1.0%).

Regarding participants’ perception of their susceptibility to HPV infection, few participants (7.2%) in this study considered themselves susceptible to contracting the disease in the nearest future which also reflected in the very low rate of participation (1.0%) in HPV testing among the sample population. This shows that an individual’s perceived susceptibility to a particular disease indeed affects whether or not they undertake preventive measures [58].

In assessing participants’ perception of the benefits of HPV testing, results show that 56.9% believed HPV testing can prevent or reduce their chances of getting cervical cancer. However, 42.9% were oblivious of the importance of HPV testing in the prevention of cervical cancer. This may be due to participants’ lack of knowledge about the causative relationship between HPV and cervical cancer. It is evident from the foregoing that lack of knowledge about HPV and HPV testing has adverse effect on an individual’s attitude towards HPV testing. Although over half of the participants understood the benefit of HPV testing, it did not positively impact their screening practice. This does not fit the theorised relationship between the concepts of perceived benefits and practice, perhaps owing to the fact that they did not believe they are susceptible to the disease. Therefore, it can be argued that a person will undertake a prescribed action if they perceive that it will be beneficial in the prevention of a disease they believe they are susceptible to. Nevertheless, there is still a need for proper education and awareness creation in this regard.

The attitude of participants who were sexually active at the time of the study was compared with that of those who were sexually inactive. From the results, it was evident that sexually inactive participants were more likely to perceive the benefits of HPV testing. This may be attributed to the fact that participants who are in a sexual relationship may have partners or significant others who deem it unnecessary to undergo cervical screening, which may in turn affect their attitude towards HPV testing.

Furthermore, the attitude of participants who had an immediate family member living with cervical cancer was compared with that of those who did not. Results show that, those who did not have a family member living with cervical cancer had a better perception of the severity of HPV than those who did. This buttresses the point earlier made, that having an immediate family member living with cervical cancer does not always translate to a good knowledge of or attitude towards the disease. Thus, amplifying the need for adequate educative campaigns on cervical cancer and HPV among individuals irrespective of their family cervical cancer history. Another reason may be that, in this study, those who have a family member living with cervical cancer were very few (n = 23) compared to those who do not (n = 363); thus, the above results may not provide the true picture of the participants’ attitude.

Regarding participants’ HPV testing practice, results show that only 1.0% had ever tested for HPV while a large percentage (98.9%) had never had one. The possible impact of self-sampling on participants’ future screening practice was also assessed. Results show that almost three-fifths (57.7%) of participants expressed willingness to undergo routine HPV test if they are allowed to collect their own samples. Although this percentage is lower than the 78% reported in a study conducted in India, Nicaragua and Uganda [15] and the 81.1% reported in another study conducted in Nicaragua [39], it is apparent that self-sampling encourages screening practice even among our sample population. On the other hand, only 37.5% of participants expressed willingness to undergo future routine HPV test even if they are not allowed to collect their samples themselves while one-fifth (20.7%) expressed unwillingness and 41.7% stated that they were not sure they will undergo routine HPV test if they are not allowed to self-sample.

### Study limitations

This study was carried out in a tertiary institution, the information obtained may not give a complete picture of what is obtainable in South Africa in general. Furthermore, there were unwillingness to participate on the part of some eligible persons and participants may withhold relevant information or give false information. This was however addressed by the protection of participants’ rights to anonymity and confidentiality by the exclusion of names or other personal information from the questionnaire, and at all times during and after the data collection process. Furthermore, self-administered questionnaires typically have a low response rate, which constituted a challenge in getting the required sample size during the data collection process.

## Conclusion

The results of this study establish a scarcity of knowledge among students regarding HPV infection, its causative relationship with cervical cancer, and vaccine availability. The implication is that students will not be inclined to carry out preventive practices such as vaccination and HPV testing, or desist from practices that leaves them vulnerable to this disease. It is therefore of utmost necessity that enlightenment programs be initiated to address this knowledge deficiency; women need to know that HPV vaccines are available and can be accessed for free. Until this is done, the fight against cervical cancer will not be complete as HPV is a major cause of cervical cancer. Furthermore, this study revealed that awareness and knowledge about self-sampling is extremely low among students. Thus, the need for awareness and educative campaigns cannot be overemphasized, and also encouragement of self-sampling for HPV testing. To encourage increased screening participation, it is imperative that students fully understand the possibility, accuracy and benefits of self-sampling.

Furthermore, introducing and encouraging students to self-sample will address the issue of embarrassment, as well as unacceptability/invasiveness of pelvic examination and attitude of healthcare workers conducting screening. Students need to understand that the benefits of screening outweigh their perceived barriers. Thus, educating students on the preventability of cervical cancer, if detected early, can help them see screening as a preventive tool rather than a death sentence—thus, addressing the issue of fear of a positive result. Proper awareness and educative campaigns can address other cited perceived barriers such as fear of pain, perception that they are too old to screen, amongst others. Although students recorded a very poor participation in screening, if proper education is given and self-sampling is encouraged and embraced, greater screening participation is achievable going forward.

## Recommendations

This study therefore recommends proper education about HPV infection, especially its causative relationship with cervical cancer, availability of HPV vaccines and where they can be accessed for free in South Africa. Furthermore, awareness creation and proper education about self-sampling, its accuracy and benefits are highly recommended in campuses. This will foster the achievement of greater screening participation among women of screening age. The awareness and educative campaigns should also be done having regard to what constitutes major sources of information for the specific population. This study further recommends that HPV testing and cervical cancer screening programs should be incorporated in campus clinics, as well as primary healthcare facilities to reach those in the grassroots. The introduction and encouragement of self-sampling in screening facilities is also recommended, with provision made for self-sampling kits and equipment necessary for handling self-collected samples.

## Data Availability

The data sheets and materials used for this study are available from the corresponding author upon reasonable request.
